# 5-HT 2A and 5-HT 2C receptor antagonism differentially modulate reinforcement learning and cognitive flexibility: behavioural and computational evidence

**DOI:** 10.1007/s00213-024-06586-w

**Published:** 2024-04-10

**Authors:** Mona El- Sayed Hervig, Katharina Zühlsdorff, Sarah F. Olesen, Benjamin Phillips, Tadej Božič, Jeffrey W. Dalley, Rudolf N. Cardinal, Johan Alsiö, Trevor W. Robbins

**Affiliations:** 1 Department of Psychology, University of Cambridge, Cambridge, CB2 3EB UK; 2 Behavioural and Clinical Neuroscience Institute, University of Cambridge, Cambridge, CB2 3EB UK; 3 Department of Neuroscience, University of Copenhagen, Copenhagen, DK-2200 Denmark; 4 The Alan Turing Institute, British Library, London, NW1 2DVB UK; 5 UCL Sainsbury Wellcome Centre for Neural Circuits and Behaviour, London, W1T 4JG UK; 6 Department of Psychiatry, Herchel Smith Building, Cambridge, CB2 0SZ UK; 7 Liaison Psychiatry Service, Cambridgeshire and Peterborough NHS Foundation Trust, Cambridge Biomedical Campus, Box 190, Cambridge, CB2 0QQ UK; 8 School of Physiology, Pharmacology & Neuroscience, University of Bristol, Bristol, BS8 1TD UK

**Keywords:** Cognitive flexibility, Reinforcement learning, 5-HT receptors, Stickiness, Reward learning, Punishment learning

## Abstract

**Rationale:**

Cognitive flexibility, the ability to adapt behaviour in response to a changing environment, is disrupted in several neuropsychiatric disorders, including obsessive–compulsive disorder and major depressive disorder. Evidence suggests that flexibility, which can be operationalised using reversal learning tasks, is modulated by serotonergic transmission. However, how exactly flexible behaviour and associated reinforcement learning (RL) processes are modulated by 5-HT action on specific receptors is unknown.

**Objectives:**

We investigated the effects of 5-HT_2A_ receptor (5-HT_2A_R) and 5-HT_2C_ receptor (5-HT_2C_R) antagonism on flexibility and underlying RL mechanisms.

**Methods:**

Thirty-six male Lister hooded rats were trained on a touchscreen visual discrimination and reversal task. We evaluated the effects of systemic treatments with the 5-HT_2A_R and 5-HT_2C_R antagonists M100907 and SB-242084, respectively, on reversal learning and performance on probe trials where correct and incorrect stimuli were presented with a third, probabilistically rewarded, stimulus. Computational models were fitted to task choice data to extract RL parameters, including a novel model designed specifically for this task.

**Results:**

5-HT_2A_R antagonism impaired reversal learning only after an initial perseverative phase, during a period of random choice and then new learning. 5-HT_2C_R antagonism, on the other hand, impaired learning from positive feedback. RL models further differentiated these effects. 5-HT_2A_R antagonism decreased punishment learning rate (i.e. negative feedback) at high and low doses. The low dose also decreased reinforcement sensitivity (beta) and increased stimulus and side stickiness (i.e., the tendency to repeat a choice regardless of outcome). 5-HT_2C_R antagonism also decreased beta, but reduced side stickiness.

**Conclusions:**

These data indicate that 5-HT_2A_ and 5-HT_2C_Rs both modulate different aspects of flexibility, with 5-HT_2A_Rs modulating learning from negative feedback as measured using RL parameters and 5-HT_2C_Rs for learning from positive feedback assessed through conventional measures.

**Supplementary Information:**

The online version contains supplementary material available at 10.1007/s00213-024-06586-w.

## Introduction

The monoamine neurotransmitter serotonin (5-hydroxytryptamine; 5-HT) system is implicated in several neuropsychiatric disorders, including major depressive disorder (MDD), obsessive–compulsive disorder (OCD) and schizophrenia, disorders in which cognitive flexibility and reinforcement learning (RL) are altered (Chamberlain et al. 2006; Clevenger et al. 2018; Zhu et al. 2021). Drugs that target the 5-HT system are often the first-line pharmacological treatment for these disorders, such as selective serotonin reuptake inhibitors (SSRIs) for MDD and OCD (APA 2010; Fineberg et al. 2020). Emerging therapies such as the 5-HT agonist psilocybin and other psychedelics are thought to hold promising treatment potential to ameliorate symptoms such as cognitive inflexibility and anhedonia (Andersen et al. 2021; Carhart-Harris and Friston 2019; Doss et al. 2021; Stroud et al. 2018). Thus, understanding the role of serotonergic modulation mediated by specific 5-HT receptors is critical for developing future therapies for disorders characterized by inflexible behaviour and diminished RL.

5-HT contributes to various cognitive processes across species, including RL (Den Ouden et al. 2013; Iigaya et al. 2018) and cognitive flexibility (Alsiö et al. 2021; Barlow et al. 2015; Clarke et al. 2004). Cognitive flexibility is defined as the ability to adapt behaviour in response to changes in the environment. Inflexible behaviour can manifest itself as compulsive behaviour, e.g. excessively perseverative actions that are independent of outcome–value associations (Berlin and Hollander 2014; Jentsch and Taylor 2001; Koob and Volkow 2016). Moreover, the ability to adjust behaviour to changes in the environment is closely linked to underlying RL processes, which integrate positive and negative feedback from the environment to maximise rewards and minimise punishment (Sutton and Barto 1998).

Flexible responding can be assessed using reversal learning paradigms across species (Uddin 2021). During reversal learning tasks, initially learned stimulus contingencies change and the subject needs to update behaviour accordingly. Substantial evidence suggests that 5-HT is involved in the modulation of reversal learning, as shown through 5-HT depletion in the orbitofrontal cortex (OFC) in monkeys (Clarke et al. 2004, 2005; Rygula et al. 2015) and rats (Alsiö et al. 2021; Izquierdo et al. 2012). In humans, acute tryptophan depletion (reducing 5-HT levels due to a reduction in its amino-acid precursor tryptophan) increases outcome-independent choice perseveration (Seymour et al. 2012) and impairs reversal learning (Kanen et al. 2021). 5-HT also modulates RL processes underlying flexible behaviour, possibly through distinct mechanisms (Bari et al. 2010; Seymour et al. 2012). In healthy human participants, short-term administration of the SSRI citalopram results in increased punishment learning and reduced reward learning (Michely et al. 2022). In patients with MDD, SSRIs impairs learning from negative feedback, while having negligible effects on learning from positive feedback (Herzallah et al. 2013). In rats, acute low-dose citalopram improves negative feedback sensitivity, while acute high-dose citalopram impairs negative feedback sensitivity, similarly to observations in human studies (Bari et al. 2010).

While it is evident that 5-HT is a key modulator of behavioural flexibility, it targets a broad range of receptor subtypes with diverse actions, exerting both excitatory and inhibitory transmission depending on receptor subtype and localisation (Alvarez et al. 2021). Thus, it is vital to understand the modulatory role of 5-HT through different receptors on cognition and RL. In particular, the excitatory 5-HT_2A_Rs, which are primarily localized on excitatory pyramidal neurons, and inhibitory 5-HT_2C_Rs, found primarily on inhibitory parvalbumin neurons, seem to be involved in reversal learning – possibly with dissociable roles (Aghajanian and Marek 1999; Amargós-Bosch et al. 2004; Liu et al. 2007; Santana et al. 2004). Systemic 5-HT_2A_R blockade impairs spatial reversal learning performance, whereas systemic blockade of 5-HT_2C_Rs improves performance (Boulougouris et al. 2008). Moreover, high levels of perseveration in rats have been found to be associated with decreased levels of 5-HT_2A_R in the OFC (Barlow et al. 2015), consistent with decreased levels of 5-HT_2A_R density in the OFC and PFC predicting clinical severity in OCD patients (Perani et al. 2008). Recent findings also suggest that psilocybin improves cognitive flexibility through a mechanism dependent on 5-HT_2A_Rs, but not 5-HT_2C_Rs (Torrado Pacheco et al. 2023). Less is known about the effects of 5-HT_2A_R and 5-HT_2C_R stimulation and blockade on component processes of reversal learning, including sensitivity to feedback and subsequent action selection.

To investigate the specific roles of 5-HT receptors in flexibility and RL, we employed the valence-probe visual discrimination (VPVD) task (Alsiö et al. 2019) and combined this task with RL modelling to gain a deeper insight into the latent processes underlying behaviour. We recently employed RL computational modelling to assess effects of 5-HT depletion and SSRI treatment in a different, probabilistic reversal task (Luo et al. 2023). We thus aimed in this study to extend this analysis to specific 5-HT receptor agents. Such models are fitted to trial-by-trial data and allow for extraction of parameters such as value-dependent (i.e., dependent on wins/losses on the previous trial) positive and negative learning rates, the ‘reinforcement sensitivity’ parameter, as well as the value-independent side and stimulus stickiness parameters, which reflect repeated responses to the same side or stimulus, respectively, regardless of the outcome on the previous trial (Daw 2009). Stickiness differs from perseveration as it provides a measure of the overall tendency to repeat a choice based on all previous trials, whereas perseveration is usually measured as the number of responses to the previously correct stimulus after a reversal. These parameters reflect different aspects of flexibility and RL, separating value-dependent from value-independent components. We examined whether these parameters contribute to choice behaviour on the VPVD task and if they were affected by 5-HT_2A_R or 5-HT_2C_R blockade. We hypothesized that 5-HT_2A_R blockade would increase stickiness parameters, and that 5-HT_2C_R blockade would lead to higher learning rates, as previous studies (summarized above) have shown increased perseveration following 5-HT_2A_R blockade and improved reversal learning behaviour resulting from 5-HT_2C_R antagonism. Computational modelling thus enables us to investigate the roles of the different 5-HT_2_ receptors more precisely in different aspects of RL behaviour.

## Materials and methods

### Animals

**Fig. 1:**
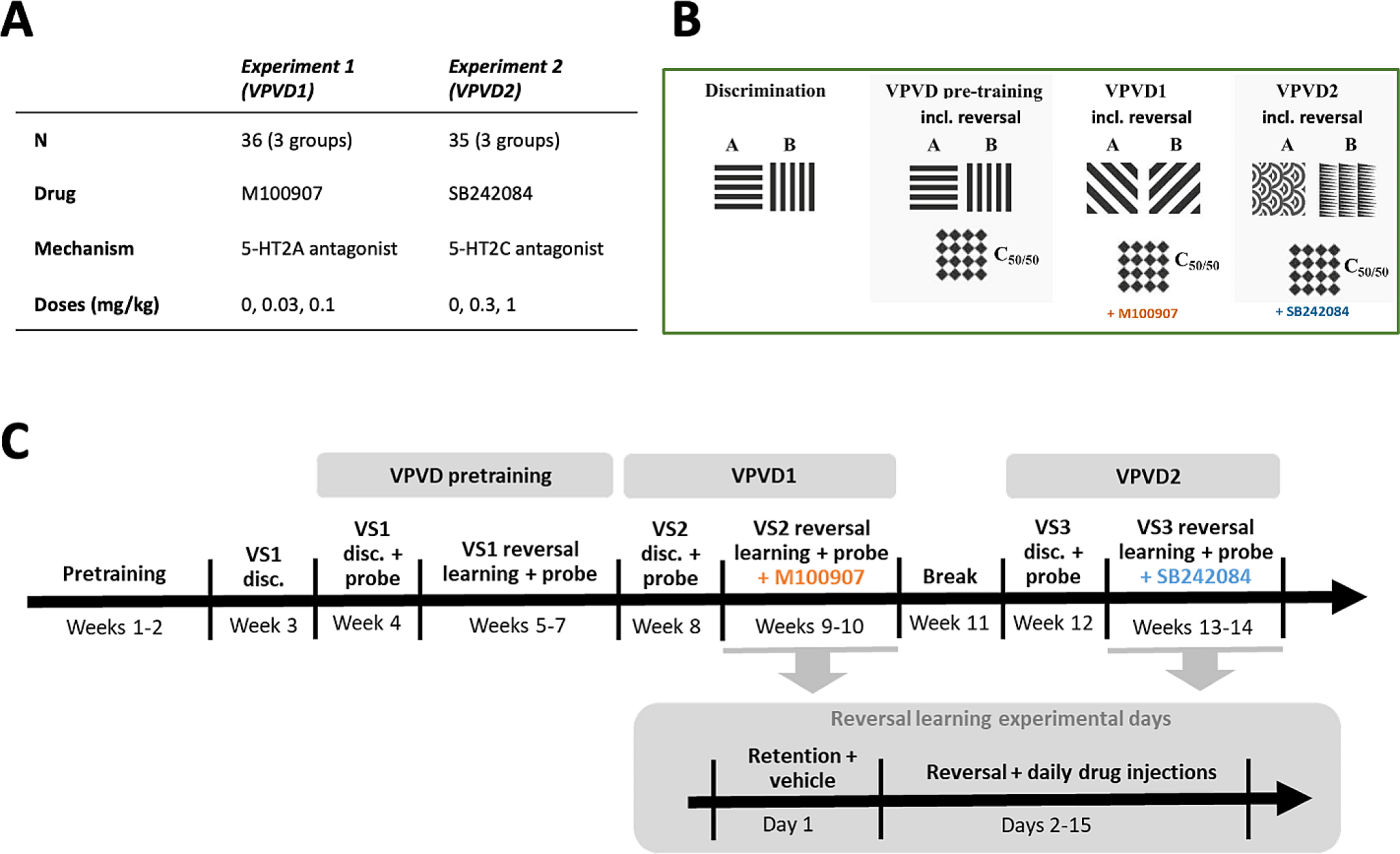
**Experimental design. (A)** Table of groups and treatments. (N, number of subjects). **(B)** VPVD stages and stimuli in the M100907 and SB-242,084 experiments. A is the 100% reinforced stimulus, B is the 0% reinforced stimulus, C is reinforced on 50% of probe trials. **(C)** Experimental timeline, including pretraining and experimental weeks. (disc., discrimination; VPVD, valence-probe visual discrimination (1 and 2); VS, visual stimulus pair (1, 2 and 3); VS2, visual stimulus pair 2; VS3, visual stimulus pair 3

Subjects were male hooded Lister rats (*N* = 36; Charles River, UK) (Fig. 1) housed in groups of three or four throughout the experiments. The rats underwent two experiments. In the first experiment (5-HT_2A_R antagonism), all 36 rats were included. In the following 5-HT_2C_R antagonist experiment, 35 rats were included, as one rat had to be euthanised due to seizures. The rats were housed under a reverse 12-h light/dark cycle with lights off at 0700 h. All training and testing was performed during the dark phase. To ensure sufficient motivation for task performance, the animals were food restricted with *ad libitum* access to water and fed once daily at random times after testing. Their body weights were maintained at 85% of their free-feeding weight. All experiments were subject to regulation by the United Kingdom Home Office (PPL 70/7548) in accordance with the Animals (Scientific Procedures) Act 1986.

### Drugs

M100907 (R-(+)-α-(2,3-dimethoxyphenyl)-1-[2-(4-fluorophenylethyl)]-4-piperidinemethanol) (Sigma Aldrich, #M3324), a highly selective 5-HT_2A_R antagonist (Kehne et al. 1996), was dissolved in 0.01 M phosphate-buffered saline (PBS) and 0.1 M hydrochloride, and adjusted with NaOH to pH 7. M100907 was administered at 0 (vehicle), 0.03 or 0.1 mg/kg.

SB-242084 (Eli Lilly, Indianapolis, IN, USA) was first dissolved in polyethene glycol 400 (PEG400) (Fisher Scientific, Loughborough, UK) at 20% of the final required volume, and then made up by 10% (w/v) hydroxypropyl-beta-cyclodextrin (Sigma-Aldrich, Poole, UK) in saline, and checked that the pH was 7. For systemic treatment, SB-242084 was administered intraperitoneally (i.p.) at doses of 0 (vehicle), 0.3 or 1.0 mg/kg in a volume of 1 ml/kg, 30 min prior to testing. Drugs were divided into the aliquots required for each test day and frozen at − 80 °C.

### Valence-probe visual discrimination task with reversal

Behavioural training was performed as previously described in (Alsiö et al. 2019). The VPVD task can assess the effect of positive or negative feedback on learning through a neutral stimulus that is probabilistically reinforced (Phillips et al. 2018). For experimental timeline and design see Fig. 1 and for additional information on the apparatus, behavioural pre-training, and touchscreen visual discrimination and reversal, see Supplementary Materials.

After pre-training, the rats progressed to the VPVD task. The VPVD task was a three-stimulus task, during which responses to one stimulus (A+) were rewarded, whereas responding to the other stimulus (B−) was punished with a time-out. A third stimulus, probabilistically rewarded on average 50% of the time (C_50/50_), was paired with either the A + or B − on ‘probe’ trials (Fig. 1).

The trial structure was kept constant, but a tone was played every time a trial was rewarded, and the stimulus duration was unlimited to ensure that animals completed the probe trials. The probe stimulus and frequency of probe trials (every 4 or 5 trials) were determined based on a previous study (Alsiö et al. 2019). After optimization, each of the probe trials was presented once every 8 trials: randomized, but never on the first trial within any 8-trial bin. There was a maximum of 200 trials per session. Both the inter-trial interval and time-out (on non-rewarded trials) were 5 s. Rats were initially tested for 5 days on the same A + and B − as during the pre-training reversal (i.e., ‘horizontal bars’ vs. ‘vertical bars’). The animals then completed a visual discrimination with a novel pair of stimuli (‘slashes’ vs. ‘backslashes’; counterbalanced across rats). Training continued for a minimum of 5 sessions but could be extended to allow rats to reach 80% correct on the standard trials within the task. Once all rats had reached the criterion, all rats progressed to the ‘reversal learning experiment’. On the day before reversal and start of drug treatment, the rats received a saline injection and were given a retention test session. The next day, rats were matched for stimulus–reward contingencies, performance on the probe trials before reversal and pre-training reversal performance, and accordingly allocated to a drug group. The stimulus–reward contingencies were reversed on the first day of reversal and then remained the same for the duration of the training sessions (i.e., there were only between-session reversals). The drug was administered before testing each day. The same stimulus (‘diamonds’) was used as the probe stimulus for all rats and across each of the phases, both during training and test trials. Training during the SB-242084 experiment followed the same procedure as above but rats were trained on a new pair of stimuli (‘arcs’ vs. ‘triangles’ counterbalanced across rats; the probe stimulus was kept the same) before reversal of the new stimulus − reward contingencies. In this case, the allocation into drug groups was also balanced based on previous drug exposure.

### Hierarchical bayesian reinforcement learning modelling

The VPVD data were modelled with RL models using a hierarchical Bayesian approach. In total, nine different models were implemented in Stan (version 2.26.1), containing different combinations of parameters. The methods and models tested are described in more detail in the Supplementary Materials.

Q-values were updated on each trial using the following equation:

1 $${Q}_{t+1}\left({c}_{t}\right)={Q}_{t}\left({c}_{t}\right)+\alpha \times \left({r}_{t}-{Q}_{t}\left({c}_{t}\right)\right)$$

where *Q*_*t*+1_(*c*_*t*_) is the Q-value of the stimulus chosen on the current trial for the next, *Q*_*t*_(*c*_*t*_) is the expected value of the stimulus selected on the current trial, *α* is the learning rate and *r*_*t*_ is the reinforcement on trial *t* (1 for reward and 0 for punishment). The learning rate reflects how much the Q-value is updated based on the prediction error *r*_*t*_ − *Q*_*t*_(*c*_*t*_), with higher α driving faster learning.

Next, the softmax decision rule was used to calculate the probability of making one of two choices:

2 $$P\left({c}_{t}=L | {Q}_{t}\left(L\right),{Q}_{t}\left(R\right)\right)=\frac{{e}^{{Q}_{t}\left(L\right)\beta }}{{e}^{{Q}_{t}\left(L\right)\beta }+{e}^{{Q}_{t}\left(R\right)\beta }}$$

Q_t_(L) and Q_t_(R) are the Q-values of the left and right stimuli, and β is the reinforcement sensitivity parameter, which determines to what extent the subject is driven by its reinforcement history (versus random choice). Lower values of β indicate greater exploration and lower sensitivity to reinforcement, whereas greater values represent increased exploitation and greater sensitivity to reinforcement.

The behavioural data were simulated with the posterior group mean parameters from the winning model, to ensure that the model could reproduce behavioural observations. The simulations were then analysed using a conventional approach as described below.

### Statistical analyses

Data across days within one reversal were collapsed, and trial outcomes were coded as perseverative, random, or learning depending on performance over bins of 30 trials in a rolling window, as described in detail and illustrated previously (Hervig et al. 2020), and following binomial distribution probabilities (Jones and Mishkin 1972).

The main measures were percentage correct responses (‘% correct’) on the standard A−< B + trials and ‘% optimal choice’ for the negative and positive probe trials across sessions. The optimal choice percentage was defined as the percentage of trials where the highest reward-probability option was chosen. Only data up to (and including) the first block of 30 trials where a rat reached criterion (24/30 correct) were analysed.

We also analysed response and collection latencies. Drug effects on standard parameters were analysed using linear mixed-effects models with the lmer package in R as described previously (Phillips et al. 2018) and as recommended for such data (Wickham 2014). The model contained two fixed factors (dose and session or dose and phase) and one random factor (subject). When relevant, further analyses were performed by conducting separate multilevel models on ‘dose’ for each session or phase. These analyses were followed by *post hoc* Dunnett’s corrected pairwise comparisons with the relevant vehicle condition. Significance was set at α = 0.05.

Visualization and statistical tests were performed with R, version 4.1.2 (R Core Team 2021). Response frequencies were square-root transformed, latencies were log transformed and probabilities were arcsine transformed to ensure normality, as confirmed with a quantile–quantile plot of residuals.

## Results

### Experiment 1: effects of systemic 5-HT_2A_R blockade on reversal learning and reinforcement learning parameters

#### Effects of systemic 5-HT_2A_R blockade on reinforcement learning processes: computational modeling

**Fig. 2:**
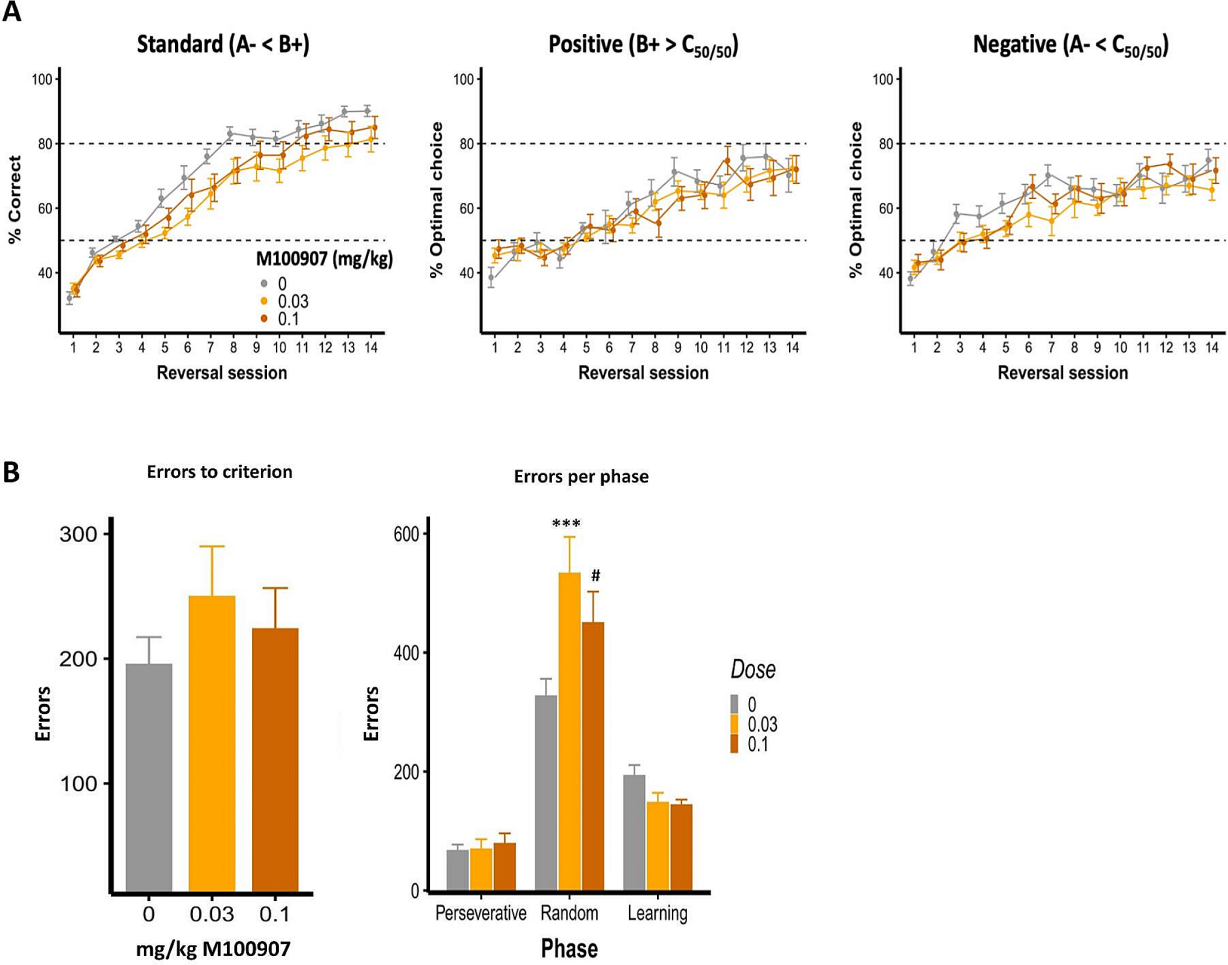
Effects of M100907 on VPVD parameters. **(A)** Percent correct and percent optimal choice across sessions. **(B)** Errors to criterion and errors per phase. Results are represented as mean ± standard error of the mean (SEM); *** *p* < 0.01, # *p* < 0.1

After computational modeling of VPVD choice behaviour, Model 9 was the best-fitting model (Table 1). This model included the following parameters: α_rew_ (reward learning rate), α_pun_, (punishment learning rate), β (reinforcement sensitivity), κ_stim_ (stimulus stickiness), κ_side_(side stickiness), and the discount factorρ. Learning from negative feedback was decreased by both low (difference in parameter per-group mean, posterior 95% highest density interval (HDI) excluding zero (group difference, 0 ∉ 95% HDI)) and high (group difference, 0 ∉ 75% HDI) doses of M100907. There was some evidence that low, but not high, dose M100907, also decreased the reinforcement sensitivity parameter (reflecting decreased sensitivity to reinforcement) (group difference, 0 ∉ 75% HDI) and increased the stimulus stickiness parameter (group difference, 0 ∉ 75% HDI). The side (location) stickiness parameter was increased in the low dose group (group difference, 0 ∉ 95% HDI) and slightly increased in the high dose group (group difference, 0 ∉ 75% HDI). The reward learning rate and discount factor were unaffected by M100907 treatment (no group differences, 0 ∈ 75% HDI) (Fig. 2and Table 2. The mean and standard deviation of the novel discount factor ρ for each group can be found in Supplementary Table 2

**Table 1:** Model comparison summary. Models were assumed to be equiprobable *a priori*

Model	Parameters	Rank (M100907)	Log marginal likelihood (M100907)	Log posterior P (M100907)	Rank (SB-248,420)	Log marginal likelihood (SB-248,420)	Log posterior P (SB-248,420)
1	α, β	9	-57308.08	-5267.60	9	-53630.42	-3381.41
2	α, β, κ_stim_	8	-57299.67	-5259.07	8	-53593.89	-3344.88
3	α, β, κ_side_	5	-52320.21	-279.61	4	-50351.08	-102.07
4	α, β, κ_side_, κ_stim_	4	-52270.79	-230.18	3	-50322.69	-73.68
5	α_rew_, α_pun_, β	6	-57182.04	-5141.43	6	-53561.99	-3312.98
6	α_rew_, α_pun_, β, κ_stim_	7	-57243.58	-5202.97	7	-53588.28	-3339.27
7	α_rew_, α_pun_, β, κ_side_	3	-52194.25	-153.64	1	-50249.01	0.000
8	α_rew_, α_pun_, β, κ_side_, κ_stim_	2	-52144.88	-104.27	5	-50386.28	-127.28
9	α_rew_, α_pun_, β, κ_side_, κ_stim,_ ρ	1	-52040.60	0.000	2	-50255.83	-6.82

**Table 2:** Summary of the effects of low and high dose M100907 and SB-242084 on reinforcement learning parameters (↑/↓, increase/decrease; - indicates no change at those levels; blank cells for parameters not tested with a given data set, as described in the Supplementary Methods). Red indicates 0 ∉95% HDI; orange indicates 0 ∉75% HDI

Parameter	M100907–0.03 mg/kg	M100907–0.1 mg/kg	SB-242,084–0.3 mg/kg	SB-242,084–1.0 mg/kg
α_rew_	**-**	**-**	**-**	**-**
α_pun_	$\downarrow$	$\downarrow$	**-**	**-**
β	$\downarrow$	**-**	**-**	$\downarrow$
κ_side_	$\uparrow$	$\uparrow$	$\downarrow$	$\downarrow$
κ_stim_	$\uparrow$	**-**		
ρ	**-**	**-**		

Furthermore, we simulated the behavioural data using the extracted parameters from the winning model. The data modelled was separated into standard, positive and negative probe trials. The simulations were able to capture the dynamics of behaviour on the VPVD task, as can be seen in the Supplementary Materials (Figure SF.1)

#### Effects of 5-HT_2A_R blockade on VPVD reversal: standard behavioural parameters

There was weak evidence that systemic M100907 impaired performance on the VPVD task. On the standard (A−< B+) trials, there was a trend towards a main effect of dose (*F*_2,35_ = 2.93, *p* = 0.066) and a trend towards a dose × session interaction (*F*_26,455_ = 1.52, *p* = 0.051) (Fig. 2A). As there were evident trending effects (although non-significant), we performed further *post hoc* analyses within each session. *Post hoc* comparisons following correction for multiple comparisons revealed that the 0.03 mg/kg dose significantly reduced correct responding on sessions 6 (*t*_112_ = -2.50, *p* = 0.027), 8 (*t*_112_ = -2.63, *p* = 0.019), 13 (*t*_112_ = -2.79, *p* = 0.012) and 14 (*t*_112_ = -2.37, *p* = 0.036). On positive and negative probe trials, we found no dose × session interactions (positive: *F*_26,455_ = 1.30, *p* = 0.15; negative: *F*_26,455_ = 1.12, *p* = 0.31) or main effect of dose (positive: *F*_2,35_ = 0.30, *p* = 0.74; negative: *F*_2,35_ = 1.52, *p* = 0.23) on % optimal choice.

For errors to criterion, there was a significant drug × phase interaction (*F*_4,105_ = 3.85, *p* = 0.0058), but no effect of M100907 overall (*F*_2,105_ = 0.21, *p* = 0.81). Further analysis based on planned pairwise comparisons showed that 0.03 mg/kg M100907 significantly increased errors in the random phase (*t*_115_ = 3.59, *p* = 0.0010), while there was a trend of 1 mg/kg M100907 towards increasing errors (*t*_115_ = 2.18, *p* = 0.060) in this phase.

### Experiment 2: effects of systemic 5-HT_2C_R blockade on reversal learning and reinforcement learning parameters

#### Effects of systemic 5-HT_2C_R blockade on reinforcement learning processes: computational modeling

**Fig. 3:**
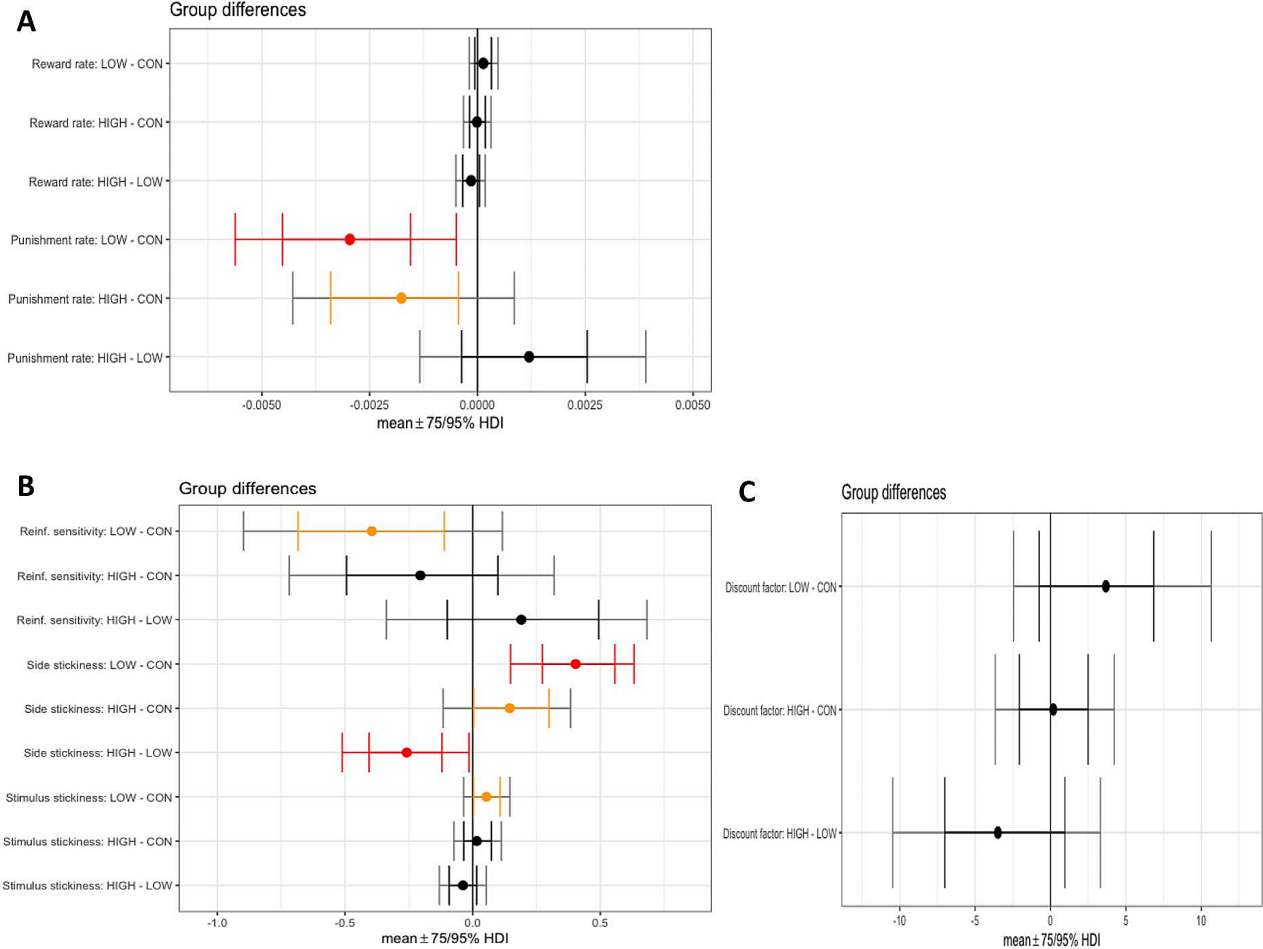
Results from the hierarchical Bayesian winning RL model 9, showing differences in group mean parameters following M100907 administration. **(A)** Reward and punishment learning rate parameters. **(B)** Reinforcement sensitivity, side and stimulus stickiness parameters. **(C)** Discount factor. (LOW, low dose; HIGH, high dose; CON, vehicle; Reinf., reinforcement; HDI, highest posterior density interval. Red indicates 0 ∉ 95% HDI; orange indicates 0 ∉75% HDI)

Model 7 was the winning model for this dataset (including parameters α_rew_, α_pun_, β and κ_side_) (Model 9 did not converge; see **Supplementary Material**). It showed that learning from positive and negative feedback were unaffected by SB-242084 (no group differences, 0 ∈ 75% HDI) (Fig. 3**and** Table 2. High-dose SB-242084 decreased the reinforcement sensitivity parameter (i.e., reducing sensitivity to feedback) (group difference, 0 ∉ 75% HDI). The side stickiness parameter was decreased by low-dose (group difference, 0 ∉ 95% HDI) and high-dose (group difference, 0 ∉ 75% HDI) SB-242,084. We also simulated the data for this experiment using the extracted parameters FigureSF.2

#### Effects of 5-HT_2C_R blockade on VPVD reversal: standard behavioural parameters

Systemic SB-242084 impaired performance in the VPVD reversal learning task. On the standard (A−< B+) trials, there was a trend towards a main effect of dose (*F*_2,35_ = 3.15, *p* = 0.055) but no dose × session interaction (*F*_26,455_ = 0.81, *p* = 0.74) (Fig. 3). On positive probe trials, there was a significant main effect of dose on % optimal choice(*F*_2,35_ = 7.38, *p* = 0.0021) but no dose × session interaction (*F*_26,455_ = 1.04, *p* = 0.41). As there were evident trending effects (although non-significant), we performed further *post hoc* analyses within each session for the standard (A−< B+) trials. *Post hoc* comparisons revealed that the 1.0 mg/kg SB-242084 significantly reduced % correct on sessions 7 (*t*_91.8_ = -2.63, *p* = 0.020) and 8 (*t*_91.8_ = -2.35, *p* = 0.040). On positive probe trials, *post hoc* analyses showed that % optimal choice was significantly decreased on sessions 8 (*t*_423_ = -2.48, *p* = 0.026), 9 (*t*_423_ = -2.61, *p* = 0.018), 11 (*t*_423_ = -2.39, *p* = 0.034) and 12 (*t*_423_ = -2.24, *p* = 0.049).

For errors to criterion, we found no effect of SB-242,084 overall (*F*_2,105_ = 1.80, *p* = 0.17). When analysing the effect of SB-242084 on errors per phase, we found a trend towards a main effect of dose (*F*_2,35_ = 3.15, *p* = 0.055) and significant effect of phase (*F*_2,70_ = 53.15, *p* < 0.0001), but no dose × phase interaction (*F*_4,70_ = 0.50, *p* = 0.73).

Win-stay/lose-shift and latency analyses for both experiments can be found in the **Supplementary Materials**.

## DISCUSSION

These findings indicated contrasting, as well as common, effects of 5-HT_2A_ and 5-HT_2C_ R antagonists on measures of RL and cognitive flexibility in the rat. We used a computational modelling approach to visual discrimination reversal that characterized novel drug effects not seen previously using standard behavioural measures. The RL parameters enabled us to gain a deeper insight into the latent mechanisms underlying behaviour on the VPVD task.

### Effects of 5-HT_2A_R antagonism on reinforcement learning and cognitive flexibility

Selective blockade of 5-HT_2A_Rs using M100907 impaired reversal learning as reflected by reductions in % correct on standard trials and an increasing frequency of errors after the initial perseverative phase at the random choice and learning phases. This impairment was not associated with changes in response or collection latencies, showing that it was unlikely to be caused by motivational or sensorimotor deficits. Computational analyses revealed that 5-HT_2A_R antagonism impaired learning from negative feedback, decreased the reinforcement sensitivity parameter and increased both side and stimulus ‘stickiness’, suggesting differential effects of 5-HT_2A_R blockade on value-dependent (reinforcement sensitivity) compared to value-independent (stickiness) choices, which may reflect distinct facets of the cognitive flexibility construct.

Previous studies using systemic (Boulougouris et al. 2008) or intra-lateral OFC (Hervig et al. 2020) M100907 have also shown impaired reversal learning performance, consistent with the present findings. Moreover, lower 5-HT_2A_R binding in the rat OFC is associated with more perseveration during spatial reversal (Barlow et al. 2015). Our findings may seem inconsistent with studies showing that the 5-HT_2A_R antagonist ketanserin normalizes impairments in flexibility resulting from lysergic acid diethylamide (LSD), which is a partial 5-HT_2A_R agonist, as well as general improvements in set-shifting following ketanserin administration in rats (Baker et al. 2011; Pokorny et al. 2020; Torrado Pacheco et al. 2023). However, such apparent inconsistencies may have resulted from the use of different paradigms to assess flexibility, such as set-shifting, which may involve distinct neural and 5-HT dependent substrates than reversal learning (Clarke et al. 2005; Dias et al. 1996).

**Fig. 4:**
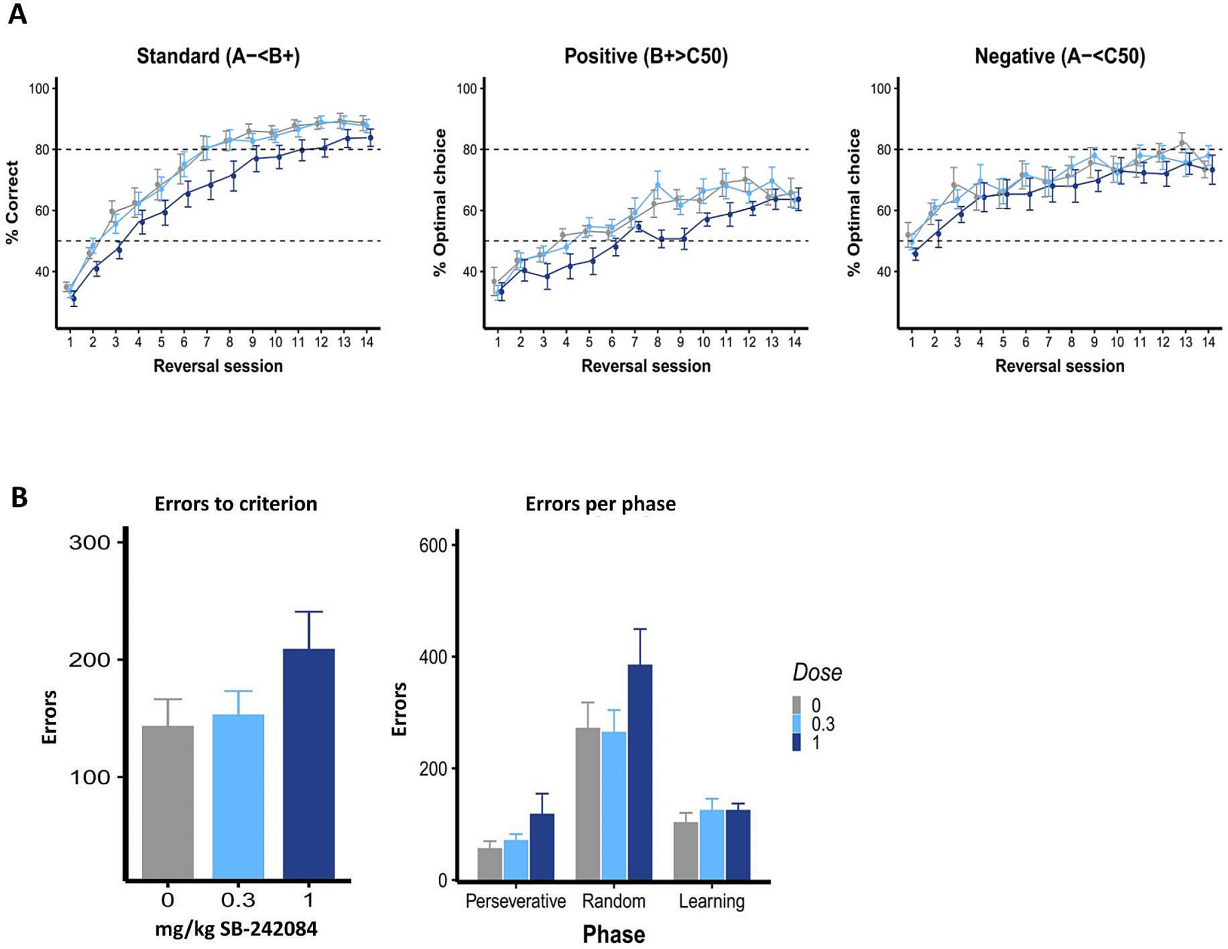
Effects of SB-242048 on VPVD parameters. **(A)** Percent correct and percent optimal choice across sessions. **(B)** Errors to criterion and errors per phase. Results are represented as mean ± SEM; *** *p* < 0.01, # *p* < 0.1

Dose may also be a relevant factor. The lower dose of 0.03 mg/kg M100907 affected reversal learning more than the 0.1 mg/kg dose, possibly reflecting an inverted U-curve effect, as previously reported for 5-HT_2A_R antagonists (Marek et al. 2005). Dose-response studies have shown that moderate systemic doses of M100907 are more effective than low and high doses on a response-inhibition task and that intra-lOFC infusions with moderate M100907 doses induce the most detrimental effects on reversal learning (Furr et al. 2012; Marek et al. 2005). The high-dose of the 5-HT_2A_R antagonist may have induced receptor internalization, an established mechanism for the 5-HT_2A_R which produces such apparently paradoxical effects (Roth 2011) (Fig. 4).

The findings align with our initial hypothesis of increased stickiness following 5-HT_2A_R blockade. Selective depletions of 5-HT in the marmoset OFC and amygdala using 5,7-DHT also results in increased side stickiness rates, similar to our findings following 5-HT_2A_R antagonism (Rygula et al. 2015), suggesting that 5-HT_2A_Rs in these areas may modulate the stickiness parameter, i.e., repeating responses regardless of previous outcomes. This accords with the demonstration that side stickiness is correlated with functional connectivity between the amygdala and medial OFC in rats (Zühlsdorff et al. 2023).

### Effects of 5-HT_2C_R antagonism on reinforcement learning and cognitive flexibility

**Fig. 5:**
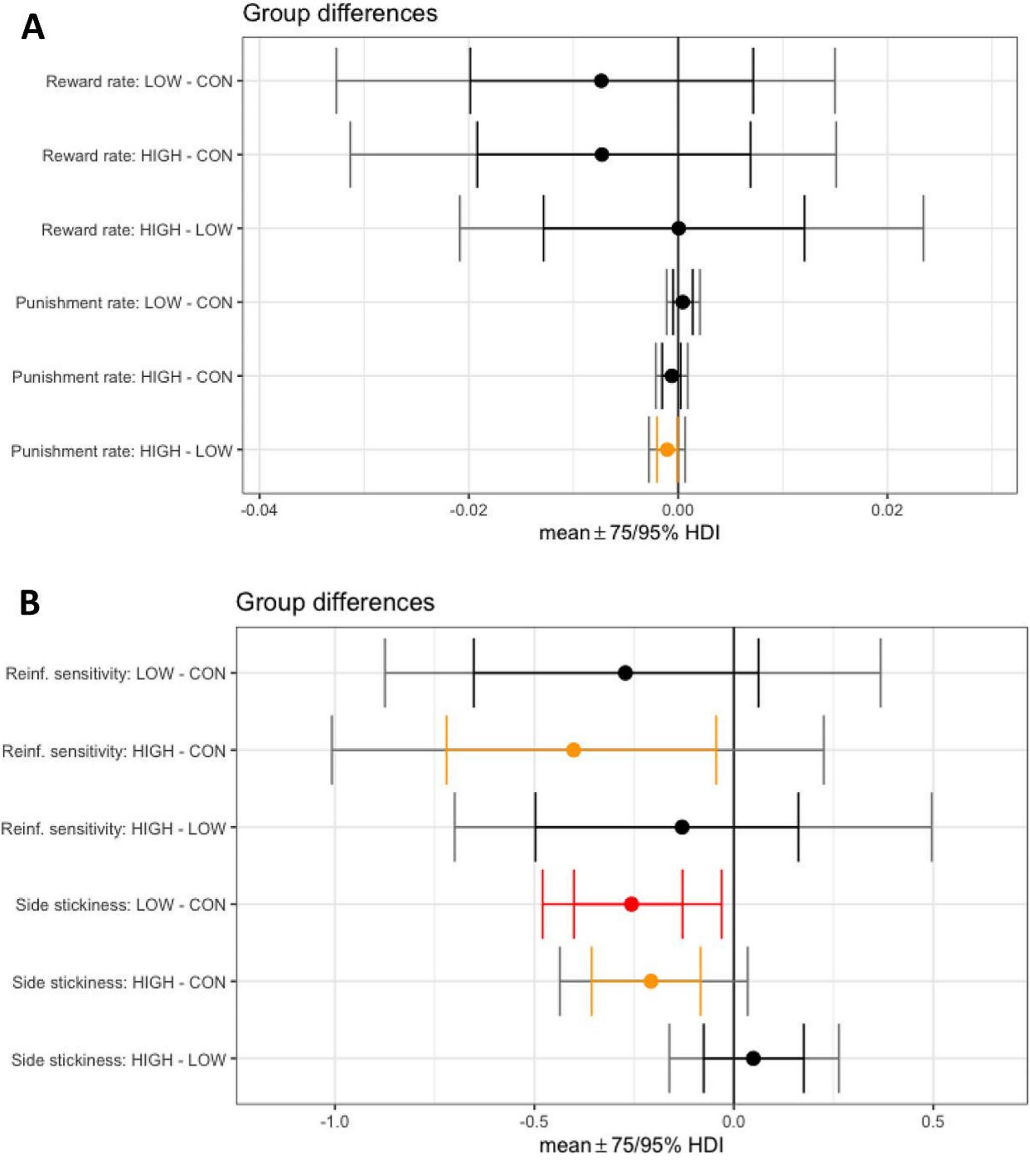
Results from the hierarchical Bayesian winning RL model 7, showing differences in group mean parameters following SB-242048 administration. **(A)** Reward and punishment learning rate parameters. **(B)** Reinforcement sensitivity and side stickiness parameters. (LOW, low dose; HIGH, high dose; CON, vehicle; Reinf., reinforcement; HDI, highest posterior interval. Red indicates 0 ∉ 95% HDI; orange indicates 0 ∉ 75% HDI)

Antagonism of 5-HT_2C_Rs with SB-242084 decreased % correct and % optimal choice on the VPVD task at high doses. Previous data have shown that this agent can improve serial reversal performance in the initial perseverative phases due to reduced perseveration but that there is an overall decremental effect on performance, possibly due to impaired (re-)learning of associations after perseveration has been overcome (Alsiö et al. 2015). This interpretation is supported by differential roles of 5-HT in lateral orbitofrontal and medial prefrontal cortex (Alsiö et al. 2019). In probabilistic reversal tasks, where there is already a high baseline of response shifting, further increases are unlikely to improve performance and may impair it (e.g., human data in (Kanen et al. 2019). Using RL models, we found here that 5-HT_2C_R blockade decreased the reinforcement sensitivity parameter at a higher dose and decreased side stickiness at low and high doses. In both the present study and in Phillips et al. (2018), SB-242084 impaired performance and reduced reinforcement sensitivity. This drug therefore appeared to enhance flexible responding as reflected by the reinforcement sensitivity and side stickiness parameters and (Fig. 5) this may account for the initial positive effects on serial reversal. This observation is in accordance with studies showing SB-242084 to improve performance during perseverative phases of serial visual reversal learning (Boulougouris et al. 2008). Our findings indicate that this improvement may be due to decreased side stickiness following SB-242084 administration. However, the reduction in reinforcement sensitivity may lead to an overall deficit in performance.

### Implications for mechanisms of action of SSRIs and psychedelics in psychiatric disorders

In a recent analysis, lower doses of the SSRI citalopram increases the reward learning rate and decreases side stickiness, whilst decreasing reward rate and increasing reinforcement sensitivity at a higher dose (Luo et al. 2023). Acute escitalopram in healthy human participants reduces the reward learning rate, decreases reinforcement sensitivity, and decreases stimulus stickiness (Luo et al. 2023), partially aligning with our findings following 5-HT_2C_R blockade. Our findings using selective 5-HT_2A_R and 5-HT_2C_R antagonists may thus aid our understanding of mechanisms underlying cognitive flexibility and RL.

Psilocybin and other psychedelics are receiving increased attention for their therapeutic potential in treating neuropsychiatric disorders such as MDD and anxiety (Carhart-Harris et al. 2016, 2021; Goldberg et al. 2020). Even though their mechanisms are poorly understood, one hypothesis is that psilocybin improves cognitive flexibility (Baker et al. 2011; Torrado Pacheco et al. 2023). Psilocybin, which primarily exerts its psychoactive effects through 5-HT_2A_R agonism (Madsen et al. 2019), has been shown to increase cognitive flexibility in individuals with MDD for at least 4 weeks (Doss et al. 2021). Ayahuasca, which contains the 5-HT_2A_R agonist dimethyltryptamine, similarly increases cognitive flexibility in healthy volunteers (Kuypers et al. 2016; Murphy-Beiner and Soar 2020). In contrast, 2,5-dimethoxy-4-iodoamphetamine, a 5-HT2_A/C_R agonist, impairs flexible strategy choice, highlighting different mechanisms of actions of hallucinogenic substances (Torrado Pacheco et al. 2023). Finally, a recent study investigating the effects on RL parameters of the psychedelic LSD, a partial 5-HT_2A_R agonist, has reported increased reward and punishment learning, and reduced stimulus stickiness (Kanen et al. 2022). Overall, these results suggest that 5-HT_2A_R agonism can improve flexibility. In the present study, we show that antagonism of this receptor decreases the punishment learning rate and increases stickiness, mirroring these hypothetical effects of 5-HT_2A_R agonism. A limitation of our study is the fact that only male animals were included; therefore, sex-dependent effects could not be investigated.

In summary, we report that both 5-HT_2A_R and 5-HT_2C_R antagonism altered performance on a visual reversal task. We characterized this impairment using RL models, finding that 5-HT_2A_R blockade reduced both learning from punishment and reinforcement sensitivity, but increased stickiness. 5-HT_2C_R blockade impaired learning from positive feedback as assessed using conventional measures, suggesting a dissociation between the two receptors: the 5-HT_2C_R is essential for learning from positive feedback and the 5-HT_2A_R is important for learning from negative feedback. Additionally, 5-HT_2C_R antagonism reduced reinforcement sensitivity and side stickiness parameters, indicating increased flexibility. These results provide novel insights into the mechanisms of 5-HT and the involvement of different 5-HT receptors in cognitive flexibility. This may be important for our understanding of neuropsychiatric conditions such as MDD and OCD, as well as for research into future treatments such as psychedelic agents that act as 5-HT_2A_R agonists.

### Electronic supplementary material

Below is the link to the electronic supplementary material.

Supplementary Material 1
